# Effective Charging of Commercial Lithium Cell by Triboelectric Nanogenerator with Ultrahigh Voltage Energy Management

**DOI:** 10.1002/advs.202404253

**Published:** 2024-06-12

**Authors:** Yiming Dai, Guoxu Liu, Jie Cao, Beibei Fan, Weilin Zhou, Yongbo Li, Jun Yang, Ming Li, Jianhua Zeng, Yuanfen Chen, Zhong Lin Wang, Chi Zhang

**Affiliations:** ^1^ School of Mechanical Engineering Guangxi University Nanning 530004 P. R. China; ^2^ Beijing Key Laboratory of Micro‐nano Energy and Sensor Center for High‐Entropy Energy and Systems Beijing Institute of Nanoenergy and Nanosystems Chinese Academy of Sciences Beijing 101400 P. R. China; ^3^ School of Nanoscience and Engineering University of Chinese Academy of Sciences Beijing 100049 P. R. China; ^4^ Institute of Intelligent Flexible Mechatronics Jiangsu University Zhenjiang 212013 P. R. China; ^5^ Center on Nanoenergy Research School of Physical Science and Technology Guangxi University Nanning 530004 P. R. China

**Keywords:** charge capacity, charging solution, lithium cell, power management system, triboelectric nanogenerator

## Abstract

It is an increasingly mature application solution that triboelectric nanogenerator (TENG) supplies power to electronic devices through its power management system (PMS). However, the previous PMS is able to manage a limited voltage magnitude and the energy storage elements are limited to capacitors. This work proposes an ultrahigh voltage PMS (UV‐PMS) to realize the charging of commercial lithium cells (LCs) by TENG. The design of UV‐PMS enables energy management of TENGs with ultrahigh open‐circuit voltages up to 3500 V and boosts the peak charging current from 30.9 µA to 2.77 mA, an increase of 89.64 times. With the introduction of UV‐PMS, the effective charging capacity of LC charged by a TENG at a working frequency of 1.5 Hz for 1 h comes to 429.7 µAh, making a 75.3 times enhancement compared to charging by TENG directly. The maximum charging power comes to 1.56 mW. The energy storage efficiency is above 97% and the overall charge efficiency can be maintained at 81.2%. This work provides a reliable strategy for TENG to store energy in LC, and has promising applications in energy storage, LC's life, and self‐powered systems.

## Graphical abstract

To achieve TENG charging of commercial lithium cells, a high‐voltage power management solution (UV‐PMS) has been developed. With the using of UV‐PMS, the peak charging current has increased of 89.64 times. The effective charging capacity of TENG per unit time has achieved a 75.3‐fold improvement compared with charging lithium cells directly, and the charging efficiency can reach to 81.2%.

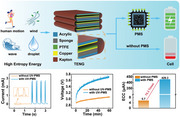

## 1 Introduction

Widely distributed IoT node sensors maintain continuous operation on the premise of long‐lasting battery life, however, the secondary maintenance of tens of thousands of energy‐supplying batteries is a great burden.^[^ ^1^, ^2^, ^3^ ^]^ Widespread high entropy energy in the environment such as wave energy, human motion energy, droplet energy, and so on are often overlooked.^[^ ^4^, ^5^ ^]^ Effective harvesting of this distributed high‐entropy energy will go a long way toward extending the limited battery life of distributed sensors and self‐powered systems.^[^ ^6^ ^]^ Theory have confirmed that triboelectric nanogenerators (TENGs), first invented in 2012, can efficiently harvest these high‐entropy energy sources.^[^ ^7^ ^]^ Compared with traditional electromagnetic generators, TENGs are more efficient at harvesting low‐frequency, low‐amplitude, and weakly input mechanical energy.^[^ ^8^, ^9^, ^10^ ^]^ In recently years, the effectiveness of TENG for harvesting a variety of energy, such as wave energy, wind energy, human kinetic energy, droplet energy, and vibration energy, have been proven.^[^ ^11^, ^12^, ^13^ ^]^ However, due to the large intrinsic matching impedance (MΩ‐GΩ) of TENG, its high output voltage (kV) and low current (nA‐µA) makes it difficult to directly drive electronic devices or supply energy to a storage device.^[^ ^14^, ^15^, ^16^, ^17^ ^]^ A power management system (PMS) is required to boost current and stabilize the output before it can provide energy for electronic devices or energy storage devices in a high‐efficiency manner.^[^ ^18^, ^19^, ^20^ ^]^ The capacitor itself suffers from fast charge release and short charge retention time, limiting its prolific use as a universal energy storage device. Lithium cells (LCs) are still widely used as energy storage units around the world due to their long power retention time, controlled discharge, and high theoretical capacity.^[^ ^21^, ^22^, ^23^ ^]^ Due to the large intrinsic internal resistance, the electrical energy output from the TENG must be first managed by the management system, then effectively charge commercial LCs.

To date, researchers have proposed a variety of power management solutions, which fall into three main categories: Travel switch,^[^ ^18^, ^24^, ^25^ ^]^ transformer^[^ ^19^ ^]^ and switching circuit.^[^ ^20^ ^]^ Most of the travel switch solutions can significantly increase the output power of the TENG. Furthermore, some switched capacitor converter solutions based on travel switches have been proposed using the principle of capacitor converter to achieve the reduction of voltage and the increase of charge.^[^ ^26^, ^27^, ^28^ ^]^ However, they are often closely related to the construction of the TENGs and are therefore not universal. Transformer solutions often demand high input frequencies, are challenging to regulate, tend to be bulky, and suffer from high transmission losses. Among the power management solutions, switching circuit solutions are widely studied for their high management efficiency and ease of regulation. The selection and design of the switch is a very critical part of the switching circuit solution. Many switching schemes have been proposed, such as spark switching schemes,^[^ ^19^ ^]^ metal‐oxide‐semiconductor field‐effect transistor (MOSFET) schemes,^[^ ^20^ ^]^ silicon‐controlled rectifier (SCR) schemes,^[^ ^29^ ^]^ and MEMS switching schemes^[^ ^30^ ^]^ Of these, SCR switches are better suited to the energy management solutions of TENGs due to their compactness, stable switching state, low transmission losses, and flexible regulation. However, TENG with higher output voltages will exceed the voltage withstand limit of a typical SCRs, besides, SCRs with withstand voltage of kilovolt tend to have larger sizes and higher losses, thus losing their advantages. Therefore, it is necessary to explore a miniaturized and efficient high voltage power management strategy.

Different from previous studies,^[^ ^15^, ^22^, ^31^, ^32^ ^]^ this work proposes an ultrahigh voltage PMS (UV‐PMS) to realize the charging of commercial LCs by TENG at the low operating frequency. The design of UV‐PMS enables energy management of TENGs with ultrahigh open‐circuit voltages up to 3500 V successfully and boosts the peak charging current from 30.9 µA to 2.77 mA, an increase of 89.64 times. With the introduction of UV‐PMS, the effective charging capacity of LC charged by a TENG at a working frequency of 1.5 Hz for 1 h comes to 429.7 µAh, making a 75.3 times enhancement compared to charging by TENG directly. The maximum charging power comes to 1.56 mW. The energy storage efficiency is above 97% and the overall charge efficiency can maintain of 81.2%. This work provides a reliable strategy for TENG to store energy in LC, has promising applications in energy storage, LC's life, and self‐powered systems. This work provides a reliable strategy for TENG to store energy in LC, has promising applications in energy storage, prolonging LC's life and self‐powered systems.

## 2 Results and Discussion

### 2.1 The Working Mechanism of UV‐PMS

**Figure 1:**
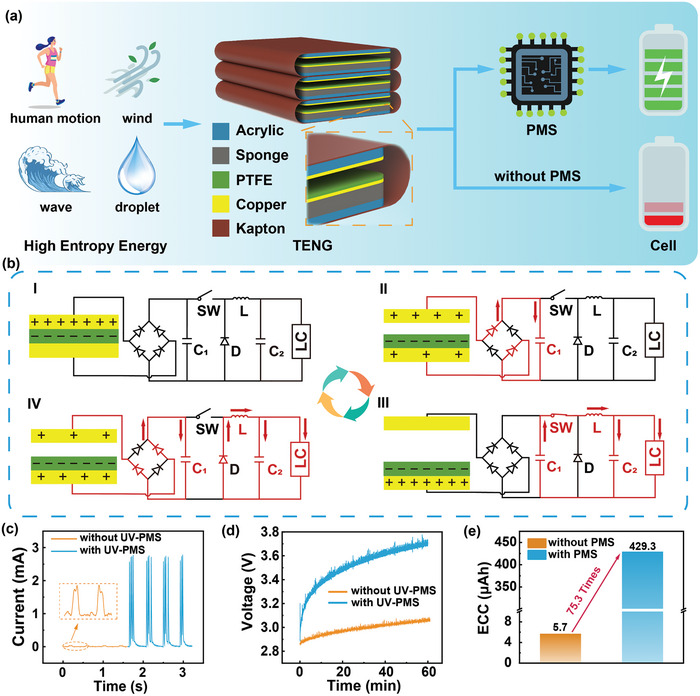
Commercial lithium cells (LCs) charging solution for TENGs. a) Overall conceptual diagram of the charging solution. b) The circuit and working process of ultrahigh voltage power management system (UV‐PMS). c) The charging current of LCs charged by TENG with and without UV‐PMS. d) The voltage variation of LIR1220 LC when charged by TENG with and without UV‐PMS. e) Comparison of the effective charge capacity (ECC) of the LC after charged by TENG with and without UV‐PMS for 1 h.

As shown in **Figure** **1**a, we construct a TENG in contact‐separation mode as the power supply, which is made up of three identical TENG units in parallel. Each TENG unit is made of a supporting layer of Kapton film, a base layer of acrylic sheet, a buffer layer of sponge, a copper film, and a polytetrafluoroethylene (PTFE) film covering the copper electrode. Among them, the copper film plays the role of the electrode and triboelectric layer, while the PTFE film acts as another triboelectric layer. The sponge is the cushioning medium to improve the contact efficiency between the two triboelectric layers. And the acrylic sheets act as support layer. The photos of TENG are shown in Figure S1 (Supporting Information). The open circuit voltage (V_OC_) of the TENG can be maintained ≈ 3500 V at operating frequency from 0.5 to 2 Hz, as shown in Figure S2 (Supporting Information). Figure 1b demonstrates the working process of the UV‐PMS. In stage I, the copper triboelectric layer comes to contact with PTFE film. At this moment, charges with opposite polarity of equal amount are induced on the surfaces of PTFE film and copper triboelectrical layer, no current is generated in the circuit. In stage II, the PTFE film begins to separate from the copper triboelectrical layer by external mechanical force and the TENG starts to generate electrical energy. Since the switch (SW) is off, the TENG charges C_1_ through the rectifier individually in this stage. As two triboelectric layers continue to separate, the TENG charges capacitor C_1_ until the voltage across C_1_ reaches the conduction threshold voltage (V_C_) of SW, triggering the UV‐PMS to enter stage III. When the SW is on, the energy stored in C_1_ is instantaneously released into inductance L, capacitor C_2,_ and LC. When the current flowing through SW is almost zero, the SW will be off implying that the UV‐PMS has entered working stage IV. At the beginning of this stage, flyback diode D starts to conduct and the energy retained within the inductance L continues to charge the LC via the flyback diode. Simultaneously, the continuous movement of the TENG results in the recharging of C_1_, initiating the subsequent operational cycle.

Here, we propose an experimental approach to describe the effective charging capacity (ECC) of LC after being charged by TENG for a period of time. Since the charging capacity of LC is difficult to measure in real‐time during the charging process by TENG, we use the discharge capacity of the LC after charging to represent the ECC of TENG. During the entire charging process, the voltage across the LC is measured and recorded in real‐time When the charging time of the LC reaches the set time, the TENG stops charging the LC and lets the LC remain stationary for 10 min. Then, the LC is discharged at a constant current of 417 µA (the integral average value of the TENG charging current) by a battery test system until the voltage reduces to the initial voltage and the voltage variation of LC and ECC during discharging are recorded. ECC is the maximum discharge capacity in each discharge progress.

Compared with charging the LC directly by TENG, UV‐PMS enhances the charging current from 30.9 µA to 2.77 mA, achieving an improvement of 89.64 times (Figure 1c). Besides, the TENG charges the LC from 2.88 to 3.7 V in 1 h with UV‐PMS, whereas the voltage could only reach 3.03 V without UV‐PMS. (Figure 1d). When charged by a TENG operating at a frequency of 1.5 Hz for 1 h, the LC's ECC increases to 429.7 µAh, implying a 75.3‐times improvement compared to charging by the TENG directly (Figure 1e).

### 2.2 The Design, Principle, and Performance of SW

**Figure 2:**
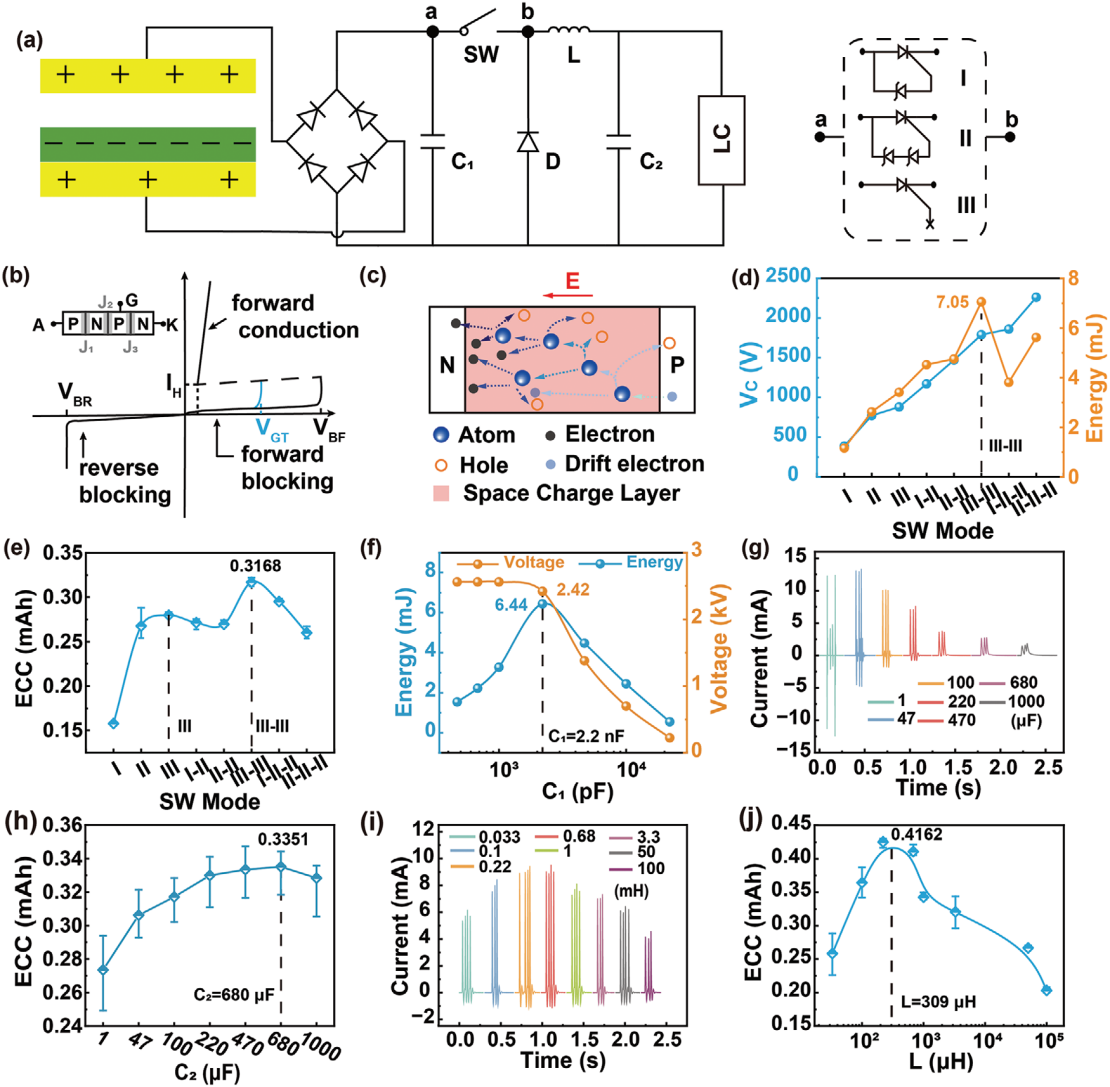
The design, principle, and optimization of UV‐PMS a) Schematic circuit diagram of UV‐PMS and silicon‐controlled rectifier (SCR) switches (SWs) in three basic modes. b) Schematic structure of SCR thyristor and the voltage‐current variation curve of the SCR working process. c) The avalanche breakdown process at the junction J_2_ of SCR. d) The conducting voltage (V_C_) of the UV‐PMS for different SW modes and the energy transferred from TENG to LC in one cycle. e) ECC of LIR1220 LC after charged by TENG using different modes of SWs for 1 h. f) The voltage across C_1_ and the stored energy varies with the value of the capacitance of C_1_. g,h) Effect of different storage capacitance C_2_ on LC charging current and ECC (mode II‐II, C_1_ = 2.2 nF, L = 3.3 mH). (i‐j) Effect of different inductors L on LC charging current and ECC (mode II‐II, C_1_ = 2.2 nF, C_2_ = 220 µF).

We propose a switch solution in the form of an SCR‐based combination, aiming at effectively regulating the ultrahigh voltage generated by the TENG. The EC103M1 SCR thyristor is selected as a switching device due to its excitation current of only 12 µA. Besides, the on‐state voltage drop of SCR has a great impact on the energy transfer efficiency. After measurement, the on‐state voltage drop of EC103M1 is only 0.67 V. The new combined switch modes consist of three basic modes enclosed within the dashed box at the right side of **Figure** **2**a. Mode I illustrates the configuration of connecting a Zener diode in reverse parallel between the anode and the gate of the SCR. The Zener diode utilized in this work is the ZY400GP with a nominal breakdown voltage of 400 V and on‐state voltage drop of 0.7 V. The operating principle of mode II is similar to that of mode I, except that the voltage load on the SW must exceed the sum of the breakdown voltages of the two Zener diodes in order to activate it. Because the breakdown voltage of commercial Zener diodes usually can't reach hundreds or even thousands of volts, connecting several Zener diodes in series with high breakdown voltage between the anode and the gate of SCR is an effective solution to improve the SCR conduction voltage. Due to the positive withstand voltage limit of the SCR, the conduction voltage of UV‐PMS can't be increased indefinitely by increasing the parallel number of Zener diodes. As for mode II, two diodes are connected in series between the anode and the gate of SCR, so the theoretical conduction voltage of mode II is 800 V. Mode III represents a special operational mode of the SCR, wherein the forward breakdown of the SCR facilitates circuit conduction, while the gate remains disconnected from any circuits or devices.

The physical model of SCR is shown in the upper left of Figure 2b, where A, G, and K are the anode, exciter, and cathode of SCR, respectively, J_1_, J_2_, and J_3_ are PN junctions. When subjected to a forward voltage, PN junctions J_1_ and J_3_ become forward‐biased, while PN junction J_2_ becomes reverse‐biased. Currently, the width of the free charge region within junction J_2_ expands, leading to an increase of the energy carried by drift charge. When the forward voltage is increased to a certain level, the energy carried by the drift charge is enough to break the covalent bond inside the free charge region and excites more electrons, resulting in avalanche breakdown (Figure 2c) and forward conduction of the SCR. The breakdown conduction time corresponds to the gate excitation conduction, which lasts for 2 ms (refers to Figure S3, Supporting Information). Since the SCR itself is self‐locking and does not shutdown actively, the conduction continues until the forward current drops to a very low value. In Figure 2b, the horizontal axis represents the voltage of the SCR, while the vertical axis represents the current passing through the SCR. The blue curve depicts the *I–V* curve of normal gate excitation conduction, V_GT_ denoting the conduction voltage. V_BF_ is the forward withstand voltage of SCR, when the forward voltage reaches V_BF_, forward breakdown conduction occurs. Hence, mode III works at the voltage of V_BF_, exceeding V_GT_, thereby the V_C_ of mode III is higher than that of mode II.

When the across voltage of SCR reaches V_BF_ in a normal direct current (DC) environment, avalanche breakdown will cause irreversible thermal damage to the junction J_2_ due to excessive current, thus making the SCR invalid. However, as for TENG, the characteristics of high voltage and low current not only enable SCR to realize the forward breakdown effect, but also avoid the problem of damaging the PN junction. Therefore, even if an avalanche breakdown occurs, the PN junction will not be damaged due to the low peak current and short peak duration. Therefore, this breakdown conduction is reproducible. Since a single SCR has a limited ability to withstand voltage, the conduction voltage of the circuit can be increased by connecting multiple basic modes in series. The cumulative conduction voltage is the aggregate of the individual conduction voltages of these modes.

In order to better demonstrate the operational stability of mode III switch, we conducted durability tests on it. As shown in Figure S4 (Supporting Information), mode III switch is able to work continuously and stably for 8 h, when the TENG is operating at a kinematic frequency of 3 Hz.

Figure 2d illustrates the V_C_ of eight modes and the corresponding energy transferred from TENG to LC in one cycle. It demonstrates that altering the mode enables the boost of V_C_. However, the energy transferred to LC does not increase with the rise of V_C_, as the peak voltage width of TENG is large, allowing SW to conduct multiple times in one cycle. The schematic diagrams, conduction times as well as conduction voltages of different modes of SWs in one cycle are shown in **Table** **1** and Figure S5 (Supporting Information). Compared with mode I‐II, the conduction numbers of SW in mode II‐II are reduced, thus the energy transferred to LC is reduced. Although the V_C_ of mode I‐II‐II is higher than that of the mode III‐III, the transferred energy becomes lower because mode I‐II‐II only conducts once per cycle. Mode II‐II‐II also conducts once per cycle with a higher V_C_ and thus more energy is transferred than mode I‐II‐II.

**Table 1:** The structures and parameters of eight modes.

SW mode	Schematic diagrams	V_C_ [V]	Conduction numbers
I	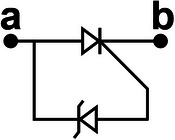	388	7
II	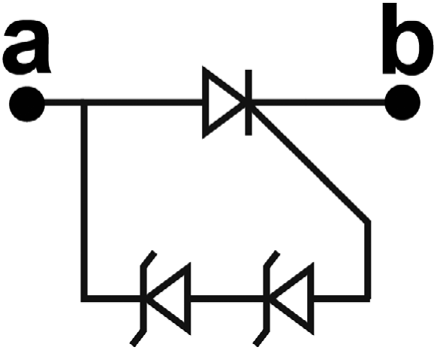	772	4
III	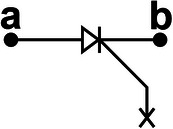	880	4
I‐II	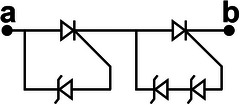	1170	3
II‐II	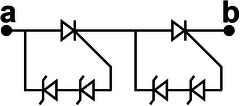	1470	2
III‐III	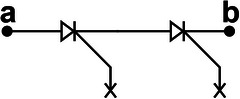	1790	2
I‐II‐II	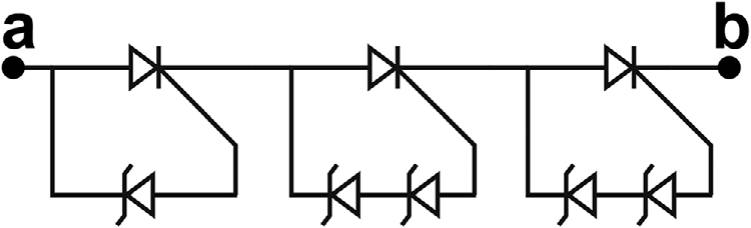	1860	1
II‐II‐II	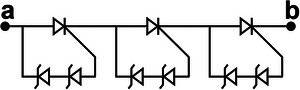	2260	1

After charging the same LC for 1 h employing different modes, the changes of ECC and the curves of discharging capacity are shown in the Figure 2e and Figure S6 (Supporting Information), respectively. Here, the ECC achieves extreme values in mode III and mode III‐III, respectively. When the V_C_ of the TENG's PMS exceeds V_BF_, the introduction of more Zener diodes will result in a decline of energy entering the LC terminal. The greater the number of Zener diodes, the greater the energy loss, consequently, less energy will transfer to the LC. Therefore, TENG with high output voltage using mode III‐III is more suitable for charging LC and maximizing energy transfer at high conduction voltage.

### 2.3 The Optimization of UV‐PMS Charging Capacity

The choice of capacitor C_1_ is important in the PMS of TENG. The summarized test results of the C_1_ matching test experiment are shown in Figure 2f. The voltage decreases gradually with the increases of C_1_, while the extracted energy reaches a maximum value (6.44 mJ) when C_1_ is 2.2 nF, indicating that the optimal value of C_1_ is 2.2 nF. In addition, the optimum value of the energy storage capacitor C_2_ in the circuit is selected, as shown in Figure 2g,h, and Figure S7 (Supporting Information). As the capacitance value of C_2_ increases, the peak charging current of the LC decreases while the peak width increases (The details of current peak width variation are shown in Figure S8, Supporting Information). In addition, the smaller the capacitance value of C_2_, the larger the reverse current, which almost disappears when C_2_ is increased to 470 µF. As the capacitance of C_2_ decreases, it experiences a faster discharge rate and exhibits a more rapid response to changes in voltage. Therefore, if the capacitance of C_2_ is small, it is not possible for LC in a short time to convert all electrical energy into chemical energy, so the voltage shows an upward trend. Besides, the LC charges the capacitor C_2_ conversely, leading to the emergence of reverse current, thereby minimizing the charging effect in practice. As shown in Figure 2h, the best matching C_2_ for charging the LC is 680 µF with the current TENG output. The optimal inductor L is selected in the same way as C_2_, as shown in Figure 2i‐j and Figure S9 (Supporting Information). There are two main effects of inductor L on UV‐PMS: on the one hand, the higher the value of inductance, the more energy will release to LC in the working phase III of the UV‐PMS. On the other hand, the effect of resistance on the circuit increases with the rise in the value of inductance. As shown in Figure 2i,j, when the inductance value is less than 220 µH, increasing the inductance value results in a higher peak charging current and a higher ECC. As the inductance value continues to increase, the influence of the resistance becomes obvious, the charging current and ECC decreases gradually. The best matching inductance is 309 µH in the curve. However, the inductor of 220 µH is used in this work because the actual ECC is larger. **Table** **2** shows the selection of electronic parameters in the optimized UV‐PMS circuit. The current measurement circuits in this work are shown in Figure S10 (Supporting Information).

**Table 2:** The selection of electronic parameters in UV‐PMS.

Electronics	Type	Parametric
C_1_	High voltage ceramic capacitors	2.2 nF
SW	Mode III‐III	1790 V
L	Power inductor	220 µH
C_2_	Electrolytic capacitor	680 µF

### 2.4 The Charging Characteristics of TENG for Charging Commercial LCs

**Figure 3:**
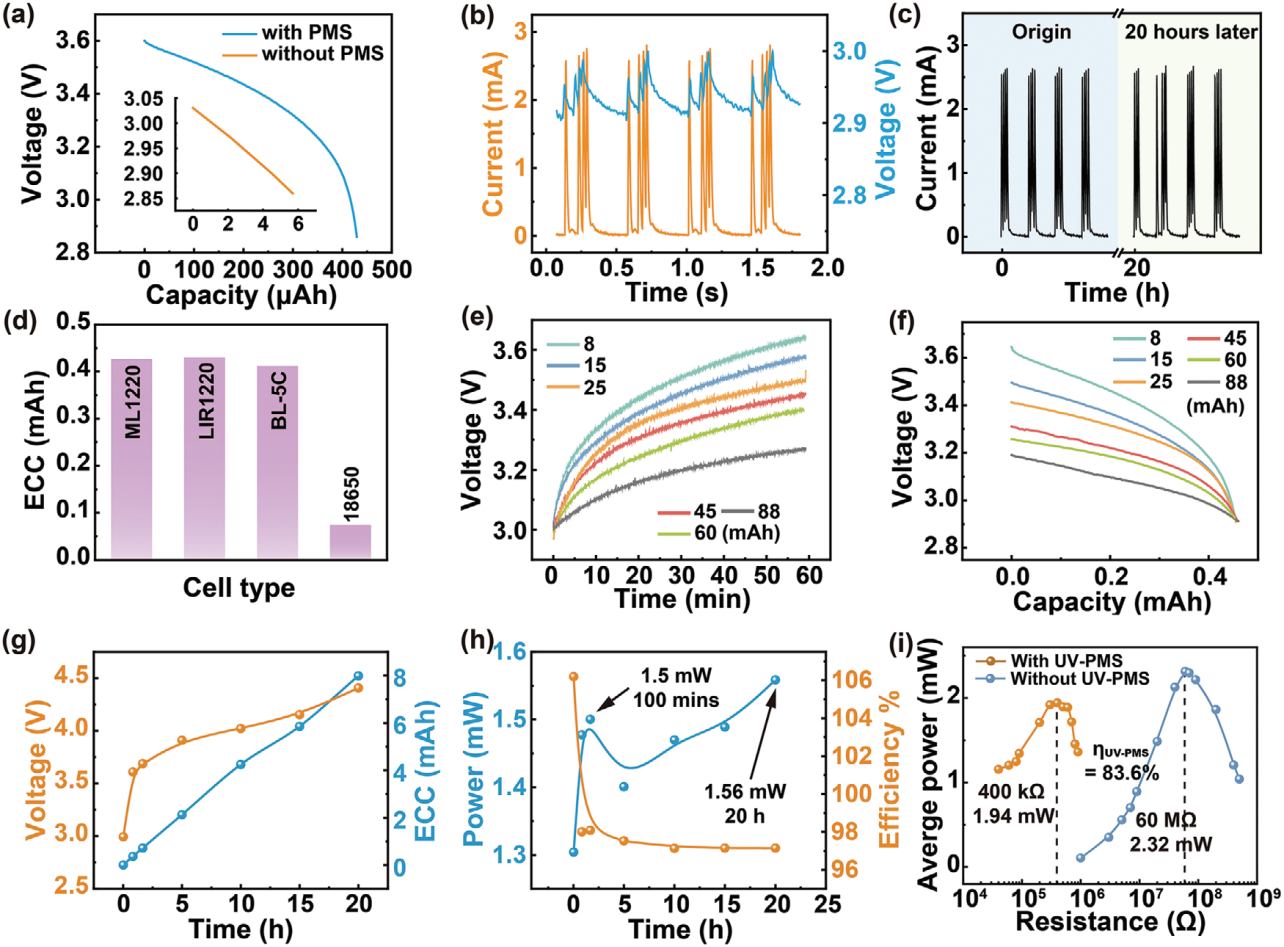
The characteristics of charging LC by TENG. a) The discharging capacity variation curves of LIR1220 LC after 1 h of charging with and without UV‐PMS. b) Variation of voltage and charging current with charging time during charging of LIR1220 LC. c). Stability characterization of charging current during LC charging by TENG with UV‐PMS. d) Summarized ECC of four different commercial LCs charged by TENG for 1 h. e) Voltage variation curves of LCs (LIR) with different capacity during TENG charging process f) Variation curves of discharge voltage with capacity of LIR series LCs with different capacities after 1 h charging by TENG. g) Variation of voltage and ECC of LC charged by TENG with charging time. h) The charging power and charging efficiency of TENG for LC. i) Output power of TENG with and without UV‐PMS and overall conversion efficiency.

The ECC of LIR1220 LC reaches to 0.4297 mAh after charged by TENG with UV‐PMS for 1 h at a working frequency of 1.5 Hz. In the absence of UV‐PMS, when LIR1220 LC is directly charged by TENG under the same experimental conditions, the ECC is only 0.0057 mAh, as shown in **Figure** **3**a. During the charging process, the peak current reaches as high as 2.77 mA, causing the LC voltage to surge at its peak, as shown in Figure 3b. After the charging current drops to 0, the potential inside the LC is rebalanced, which shows the decrease of external voltage. With the continuous input of pulse current, the LC continuously repeats the reaction‐equilibrium process to store energy, so that the LC voltage shows a wave‐like rise during the charging process. Furthermore, the stability of the charging current is tested, and after 20 h of charging, the charging current remains virtually unchanged, as shown in Figure 3c. The ECC of four common commercial LC charged by TENG for 1 h is also tested. The parameters of four LCs are shown in **Table** **3**. As shown in Figure 3d, the LIR1220 LC exhibits the best charging effect with a ECC of 0.4293 mAh, and the ML1220 LC and BL‐5C LC exhibit a slightly worse charging effect than the LIR1220 LC, whereas the 18 650 LC has a ECC of only 0.0741 mAh, which may be due to the higher requirement of charging current of the 18 650 LC. The physical photos of the four commercial LCs are shown in Figure S11 (Supporting Information).

**Table 3:** Parameters of four common commercial LCs.

LC name	Normal voltage [V]	Normal capacity [mAh]
ML1220	3	8
LIR1220	3.6	8
BL‐5C	3.6	1020
18650	3.6	1500

Figure 3e illustrates the voltage variation curves of LCs with different nominal capacities charged by TENG with UV‐PMS for 1 h. As the LC capacity increases, the growth rate of LC voltage decreases. Conversely, the lower the nominal capacity, the less time it takes to charge the LC to its operating voltage. Figure 3f illustrates the voltage‐capacity curves of LCs with different normal capacities during the constant current discharge process after being charged by TENG for 1 h. Although the growth rate of voltages is different, the ECC of different LCs is almost the same. In addition, the BL‐5C voltage changed only 0.03 V after charged by TENG for 1 h, but the discharge capacity was 0.4287 mAh, which is very close to that of the other LCs (Figure S12, Supporting Information). Therefore, different from capacitors, for many kinds of LCs, especially LIR series LCs, the nominal capacity does not have a significant effect on the charging capacity.

As shown in Figure 3g, during the charging process of the LC, the voltage growth rate gradually slows down, that is accelerated when it is close to being full, which coincides with the voltage variation curve of the LC in the process of constant current charging. Unlike the voltage variation curve, the charging capacity basically increases linearly owing to the almost constant peak current during the whole charging process, which is different from the charging characteristic of capacitors.

The actual charging power as well as the charging efficiency of the TENG for charging LCs are also investigated. The method is to charge the same LIR1220 LC from 2.5 V for 1, 50, 100, 300, 600, 900, and 1200 min, respectively. Then the LC is discharged at constant current of 417 µA until the voltage dropped to 2.5 V using the battery test system, respectively, and record the variation of voltage and discharge capacity. Finally, the effective charging powers under different charging durations are calculated by Equation (1).

(1) $$P_{C}=\frac{W_{C}}{t}=\frac{\int V_{C}\left(t\right)\times I_{C}\left(t\right)dt}{t}=\frac{\int V_{C}\left(t\right)\times d\left(\int I_{C}\left(t\right)dt\right)}{t}$$

where *P_C_* is the charging power, *W_C_* is the energy input to the LC over a period of charging time, *V_C_* is the voltage of the LC during charging process, *I_C_* is the charging current, *t* is the charging time of the LC. To more accurately represent the actual power during charging, we use the energy released from the LC during discharging process to replace the energy input to the LC over a period of charging time. Then the Equation (1) can be changed to

(2) $$P_{c}=\frac{W_{d}}{t}\;=\frac{\int VdC}{t}\;$$

where *W_d_* is the energy released from the LC during discharging process, *V* is the voltage of the LC during discharging process, *C* is the capacity of the LC during discharging process. Figure 3h shows that the charging power grows rapidly in the first 50 min, whereas, the charging power decreases to ≈ 1.4 mW after 100 min of charging, and then continues to rise until it is full. The maximum charging power can reach to 1.56 mW. There are two reasons for the characteristics of the effective charging efficiency curve:

First, as shown in Figure 3g, the voltage and capacitance of LC both increase with the extension of charging time, especially the capacity of LC shows a liner rising with charging time. Thus, the integral ∫VdC increases with the charging time and the *P_C_* shows an upward trend too. The voltage‐capacity curves of LC during discharging processes are shown in Figure S13 (Supporting Information).

Second, it can be observed from the voltage variation curve that the voltage of LC exhibits an inflection point, with a rapid rate of voltage change preceding it and a slower rate following it. Moreover, the ability of LC to maintain voltage during discharge gradually increases with charging time. Consequently, when the charging time is less than 100 min, the voltage‐capacity curves during discharging process drop rapidly and $\frac{\int VdC}{t}$ shows a rapid upward trend due to the rapid rise of the voltage of LC. As the charging time increases, the rising rate of LC voltage slows and the ability of the LC to maintain voltage during discharging process increases. The rising of ∫VdC begins to be influenced primarily by the ability of the LC to maintain voltage and starts to slow down as the charging time *t* increases. Accordingly, there is a declining stage in the power curve.

Different from the characteristics of capacitors storing energy, there are inflection points in the charging power variation curve of TENG.

The energy storage efficiency is calculated by Equation (3)

(3) $$\mu_{ES}=\frac{ECC}{\int I_{c}\left(t\right)dt}\;$$

Where, µ_*ES*_ is the energy storage efficiency, *ECC* is the effective charging capacity, *I_c_* is the charging current, *t* is the charging time.

As shown in Figure 3h, energy storage efficiency of TENG gradually decreases with the increase of charging time, but it basically remains above 97%. Since the battery is not discharged sufficiently before charging, the battery contains residual charge inside, and when the battery is discharged again after charged by TENG, the discharged capacity is the sum of the charge provided by TENG and the residual charge in battery. At the initial stage, the residual charge is a relatively large proportion of the charge provide by TENG due to the short charging time, which leads to the ECC is higher than $\int I_{c}\left(t\right)dt$ and the energy storage efficiency is higher than 100%. As the charging time increases, the effect of the residual charge decreases, the energy storage efficiency tends to stabilize. Consequently, the variation trend of the curve in Figure 3h is high at the beginning of the charging phase, decreases rapidly as the charging time increases, and gradually stabilizes around a value later.

It should be noted that the energy storage efficiency µ_*ES*_ is not the overall charging efficiency. The overall charging efficiency should include the energy management efficiency of the UV‐PMS and the energy storage efficiency of LC, the calculation is shown in Equation (4)

(4) $$\mu_{C}=\mu_{ES}\;\times \mu_{UV-PMS}$$

where, µ_*C*_ is the overall charging efficiency, µ_*ES*_ is the energy storage efficiency, µ_*UV* − *PMS*_ is the energy management efficiency of the UV‐PMS and the value of µ_*UV* − *PMS*_ is 83.6% as shown in Figure 3i. Consequently, the overall charging efficiency is 81.2%.

In addition, the charging characteristics of ML1220 LC are also tested, and the results are consistent with LIR1220 LC, as shown in Figure S14 (Supporting Information).

Moreover, lithium dendrite is often a primary cause of damage to lithium batteries, and pulse charging has been identified as a approach to suppress such growth.^[^ ^33^, ^34^ ^]^ However, the charging current produced by the TENG and enhanced through UV‐PMS differs from traditional pulse charging methods due to its low‐duty cycle and non‐constant nature. Consequently, scanning electron microscope (SEM) images of the anode of ML1220 LCs under different cycling conditions are captured as shown in Figure S15 (Supporting Information). Compared with constant current, the lithium deposition on the anode surface after charging by TENG for one cycle presents a cluster shape, which is flatter than the dendritic shape of constant‐current charging. With the increase of recharge cycles of lithium cell by TENG, the cluster‐like group formed by lithium deposition on the anode surface gradually grows up, and after 10 cycles of recharge, the cluster‐like group connects into a piece to become a moss‐like structure. However, the constant current recharge cycle to the fifth time, has produced obvious dendritic structure, after 10 times of constant current charging, the anode surface has been widely distributed spiky lithium deposits. Therefore, TENG can effectively inhibit the generation of lithium dendrites to improve the life of LC.

### 2.5 Demonstration of Applications

**Figure 4:**
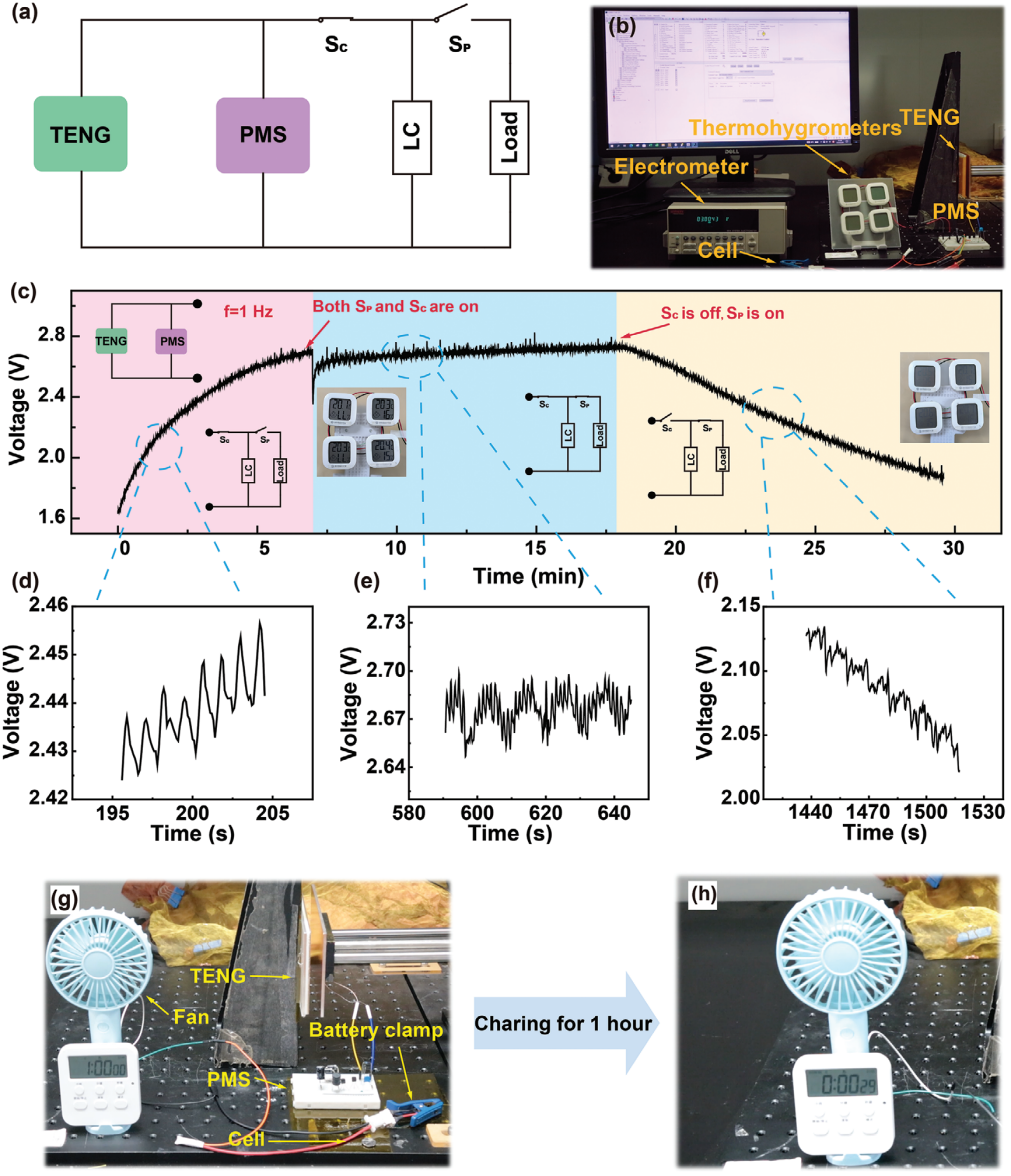
The effective charging of LC by TENG facilitates the stable and prolonged operation of electronic devices. a) Application demonstration for LCs powered by TENG. b) Photo of a TENG charging a LIR1220 LC and successfully subsequently powering four thermohydrometers. c) The voltage waveforms of the LIR1220 LC during the charging process by TENG and. d–f) Details of the voltage variation of LIR1220 LC in three application scenarios. g,h) A small fan is driven by the ML1220 LC after charged by TENG for 1 h.

Here, several applications are conducted to demonstrate the efficient charging of LCs by TENG and the stable operation of electronic devices powered by TENG. As shown in **Figure** **4**a, we can realize three application modes via introducing two switches: TENG charging the LC alone, TENG powering electronic device continuously, and the LC powering the electronic device alone. Switch S_C_ controls the charging of the LC and switch S_P_ controls the powering of the load. When S_C_ is on and S_P_ is off, it enters the LC charging mode. When S_C_ and S_P_ are both on, it enters the continuous power supply mode. When S_C_ is off and S_P_ is on, it enters the LC powering alone mode.

In the mode of LC charging, a single TENG charged the LIR1220 LC from 1.6 to 2.7 V within 7 min at the working frequency of 1 Hz. Subsequently, the S_P_ is on, demonstrating the continuous working of thermohydrometers powered by TENG. At the beginning, the voltage of LC drops immediately, then quickly stabilizes ≈ 2.7 V. At the working frequency of 1 Hz, the TENG stably supplied power for four thermohydrometers and the voltage fluctuation is only 0.06 V. Finally, when S_C_ is disconnected and the charged LC is only used to drive the thermohydrometers, the voltage of LC can still keep above 2 V for 10 min (Video S1, Supporting Information).

In order to reflect the charging ability of this charging solution more intuitively, an ML1220 LC is also charged by TENG for 1 h, and then the LC is connected to a small fan. The small fan is successfully powered by the LC charged by TENG. (Figure 4g,h; Video S2, Supporting Information).

## 3 Conclusion

Conventional energy management systems are often difficult to manage the energy of TENGs with ultra‐high V_OC_ and low working frequencies. In addition, they tend to use capacitors to store the energy from TENGs, which is not conducive to the stable operation of electronic devices. In this study, a UV‐PMS designed for charging LCs using TENG is developed, achieving a successful management of ultra‐high voltages of up to 3500 V with a simple structure and convenient adjustment. After power management, the peak current for charging LCs increased from 30.9 µA to 2.77 mA, an increase of 89.64 times. With the access of UV‐PMS, TENG can increase the charging capacity of LCs by ≈ 75.3 times at a working frequency of 1.5 Hz. Different from capacitor, the nominal capacities of LCs have little effect on the charging capacity, nevertheless the voltage rise rate during the charging process increases as LC nominal capacity decreases. The variation of LC capacity during charging exhibits linear growth, while the charging power shows an increasing trend with turning points. The charging power can reach to 1.56 mW during the process of fully charging the LC. Moreover, the energy storage efficiency gradually tends to stabilize above 97% and the overall charge efficiency can maintain of 81.2%. In the applications experiment, TENG can charge LC efficiently and power the thermohydrometers stably. This work proposes a practical, efficient and flexible TENG power management system for TENG with ultrahigh voltage to charge LCs, which shows a broad prospect in energy storage, LC life, and self‐powered systems.

## 4 Experimental Section

### Fabrication of the Three‐Layer TENG

The TENG made in this work is in contact‐separation working mode, and the electrode plate area is 143.97 cm^2^. The 4 mm thick acrylic board was cut into two rounded rectangles with a length of 16 cm and a width of 9 cm by a laser cutter (the radius of the rounded corners was 1 mm). Based on acrylic, one of them was covered with a sponge with a thickness of 3 mm, a copper film with a thickness of 49 µm was laid on the sponge as a metal electrode, then a PTFE film with a thickness of 49 µm was stuck on the copper film as a dielectric layer. The electrode plate was polarized at a high voltage of 7 kV for 10 min. On the other electrode plate, a 49 µm thick copper film was pasted on the acrylic substrate. After that, these two electrode plates were packaged by a Kapton film (200 µm) to form a single TENG. Finally, these three identical TENGs were connected in parallel.

### Construction of UV‐PMS

UV‐PMS used rectifier diodes (model 2CL71) as the rectifier bridge, high‐voltage ceramic capacitor as C_1_, EC103M1 as SCR model, MUR460 as the flyback diode, power inductor as the inductor L and electrolytic capacitor as the capacitor C_2_.

### Measurement

The linear motor was used to control the contact‐separation motion of TENG. The output performance of the TENG, as well as the voltage across components in the UV‐TENG and the voltage of the LC, were measured using an electrometer (Keysight 6514), electrostatic voltmeter (Trek 347), and oscilloscope (Tektronix MDO 3024). LANHE battery test system was used for constant current discharge test of LCs.

## Conflict of Interest

The authors declare no conflict of interest.

## Supporting information

Supporting Information

Supplemental Video 1

Supplemental Video 2

## Data Availability

Research data are not shared.
