# Supplementary material for: Effective Charging of Commercial Lithium Cell by Triboelectric Nanogenerator with Ultrahigh Voltage Energy Management
Source: Adv Sci (Weinh). 2024 Jun 12;11(30):2404253. doi: 10.1002/advs.202404253 (PMC11321660; doi:10.1002/advs.202404253)
Supplement: Supplementary file 1 — Supporting Information [file ADVS-11-2404253-s001.docx]

**Supporting Information**

Effective Charging for Commercial Lithium Cell by Triboelectric Nanogenerator with Ultrahigh Voltage Energy Management

*Yiming Dai^1, 2, #^, Guoxu Liu^2, 4, #^, Jie Cao^2, 3^, Beibei Fan^2, 5^, Weilin Zhou^1, 2^, Yongbo Li^1, 4^, Jun Yang^2,5^, Ming Li^1, 2^, Jianhua Zeng^2, 5^, Yuanfen Chen^1,^ *, Zhong Lin Wang^2, 4,^ *, Chi Zhang^1,^ ^2, 4,^ **

^1^ School of Mechanical Engineering, Guangxi University, Nanning, 530004 China

^2^ Beijing Key Laboratory of Micro-nano Energy and Sensor, Center for High-Entropy Energy and Systems, Beijing Institute of Nanoenergy and Nanosystems, Chinese Academy of Sciences, Beijing 101400, P. R. China

^3^ Institute of Intelligent Flexible Mechatronics, Jiangsu University, Zhenjiang 212013, China

^4^ School of Nanoscience and Engineering, University of Chinese Academy of Sciences, Beijing 100049, P. R. China

^5^ Center on Nanoenergy Research, School of Physical Science and Technology, Guangxi University, Nanning 530004, P. R. China.

^#^ These authors contributed equally to this work.

* Authors to whom correspondence should be addressed.

E-mail addresses: yuanfenchen@gxu.edu.cn (Y.F. Chen); zlwang@gatech.edu (Z.L. Wang); czhang@binn.cas.cn (C. Zhang)

**Supporting Figures:**

Figure S1: The photographs of the three-layer TENG.

Figure S2: The open circuit voltage of TENG.

Figure S3: The conduction time of SW.

Figure S4: Durability testing of mode III switch.

Figure S5: The voltage of C_1_ in UV-PMS with different SW modes.

Figure S6: The discharge voltage-capacity variation curve and error interval of LIR1220 after charged by TENG through UV-PMS with different SW modes for one hour.

Figure S7: The discharge voltage-capacity variation curve and error interval of LIR1220 after charged by TENG through UV-PMS with different capacitors C_2_ for one hour.

Figure S8: Details of peak current width variation for states with different values of C2.

Figure S9: The discharge voltage-capacity variation curve and error interval of LIR1220 after charged by TENG through UV-PMS with different inductances L for one hour.

Figure S10: Photos of batterie (a) LIR1220, (b) ML1220, (c) BL-5C and (d) 18650.

Figure S11: The charging current measurement circuit.

Figure S12: The voltage-time variation during charging process and the discharging capacity-time variation curve after charged by TENG of BL-5C LC.

Figure S13: The voltage-capacity variation curves of LIR1220 LC during discharging process after different charging durations.

Figure S14: Charging characteristics of ML1220 LC.

Figure S15: Scanning electron microscope (SEM) images of ML1220 LC anode.

**Supporting videos:**

Video S1: A TENG charging a LIR1220 LC and successfully subsequently powering four thermohydrometers.

Video S3: A small fan is driven by the ML1220 LC after charged by TENG for one hour.

**
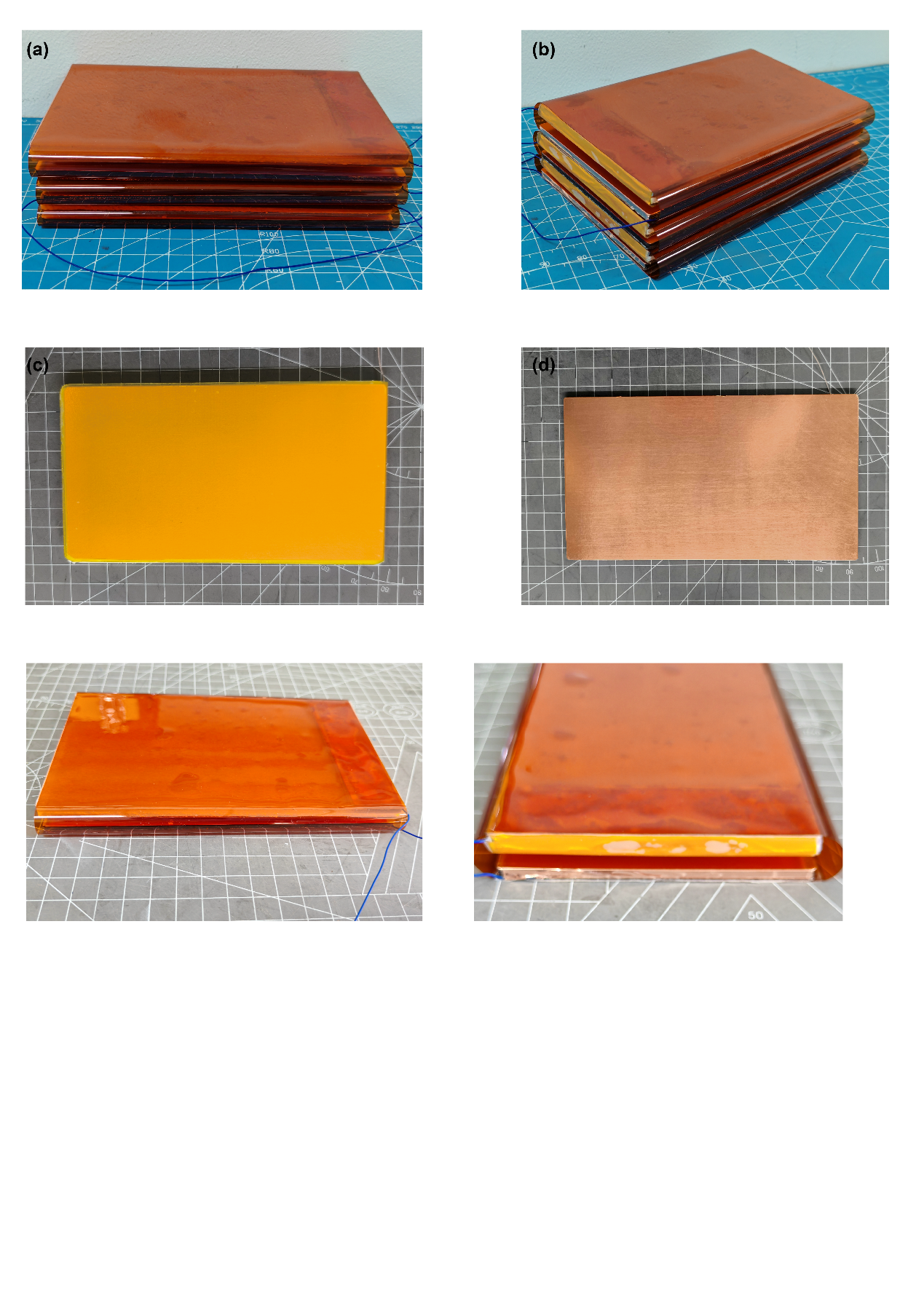
**

**Fig. S1** The photographs of the three-layer TENG. (a-b) The Photographs of the three layers TENG. (c) The upper electrode plate of a single TENG. (d) The lower electrode plate of a single TENG.


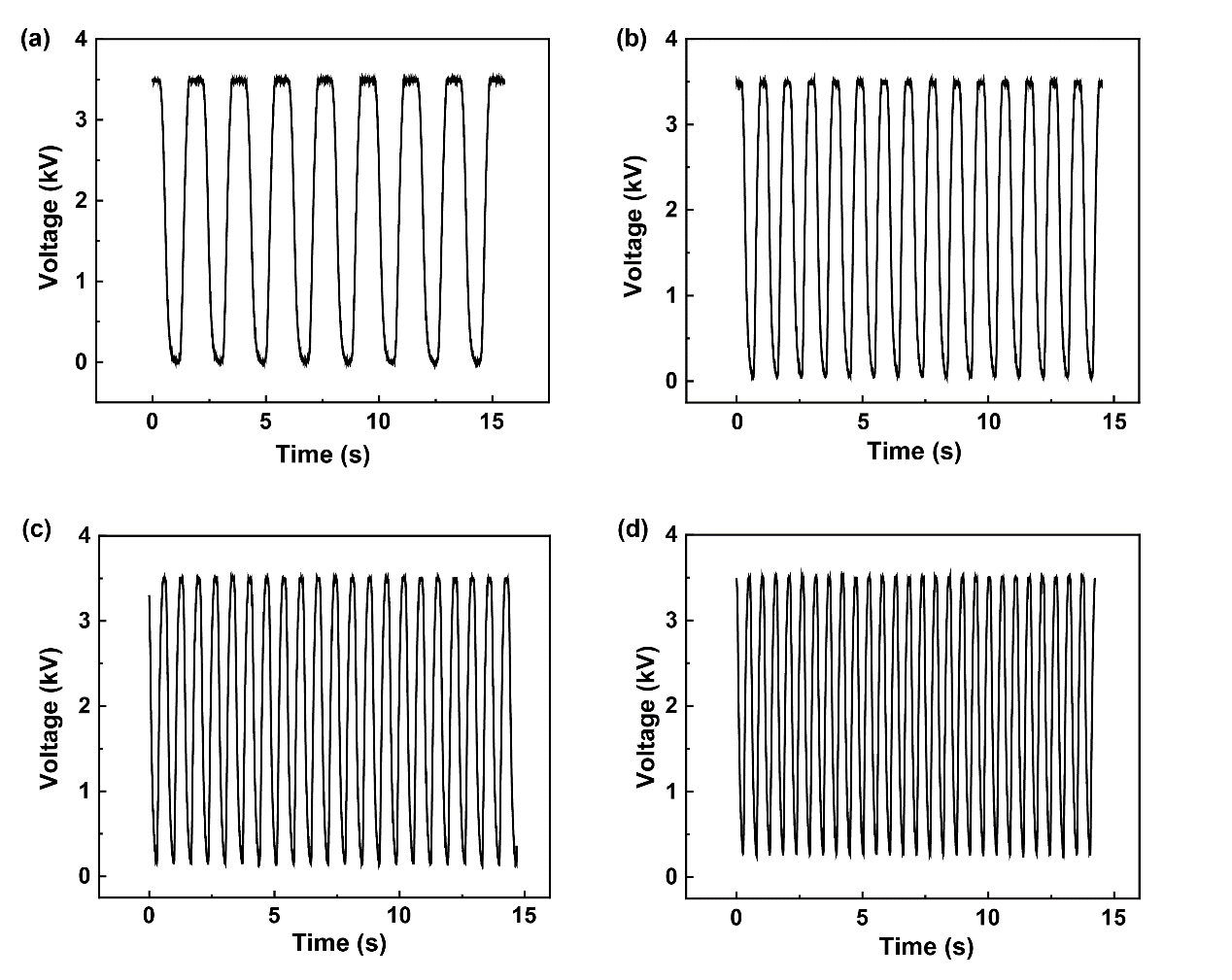


**Fig. S2** The open circuit voltage of TENG at (a) 0.5 Hz, (b) 1 Hz, (c) 1.5 Hz, (d) 2 Hz.


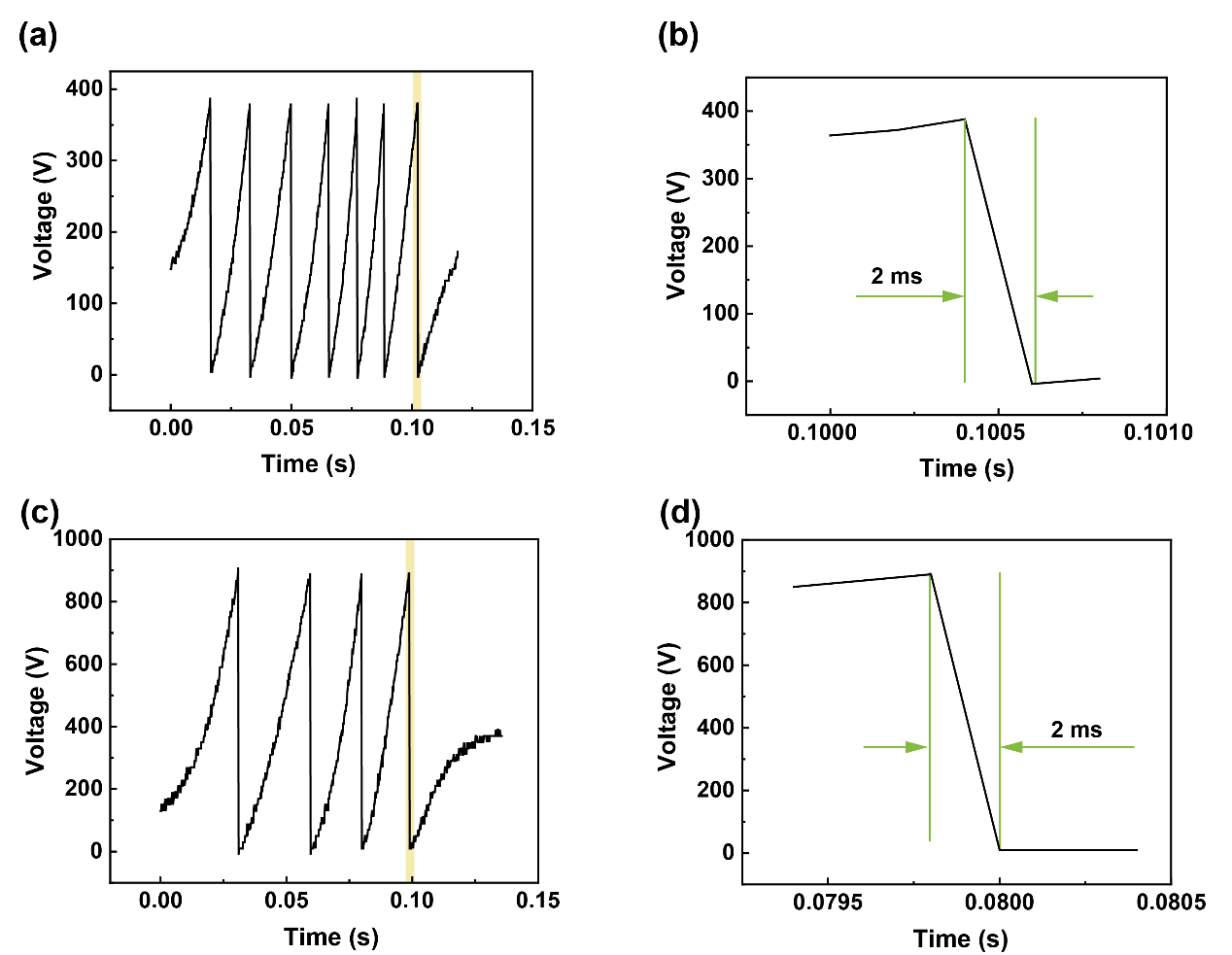


**Fig. S3** The conduction time of SW (a-b) mode I and (c-d) mode III.


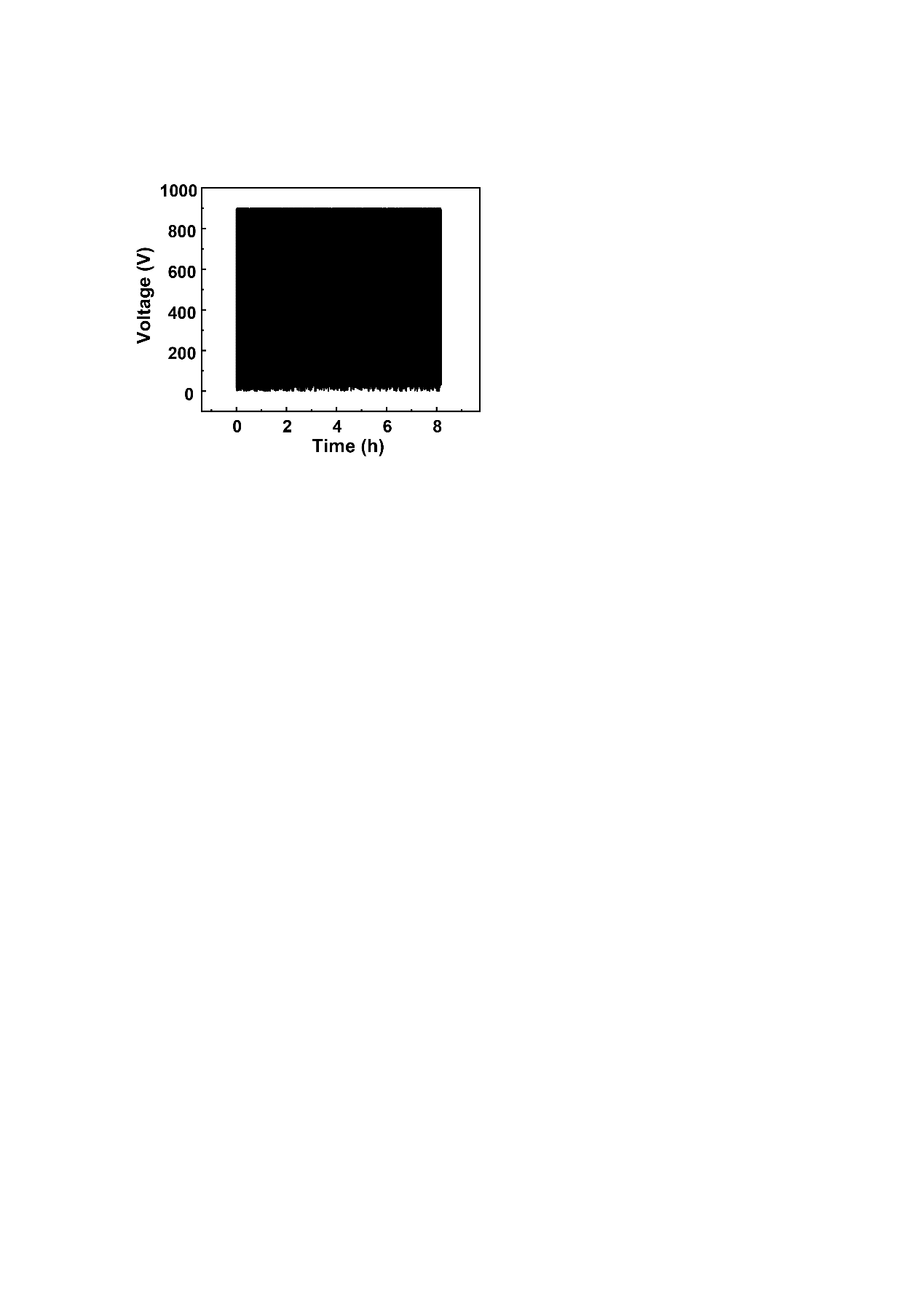


**Fig. S4** Durability testing of mode III switch.


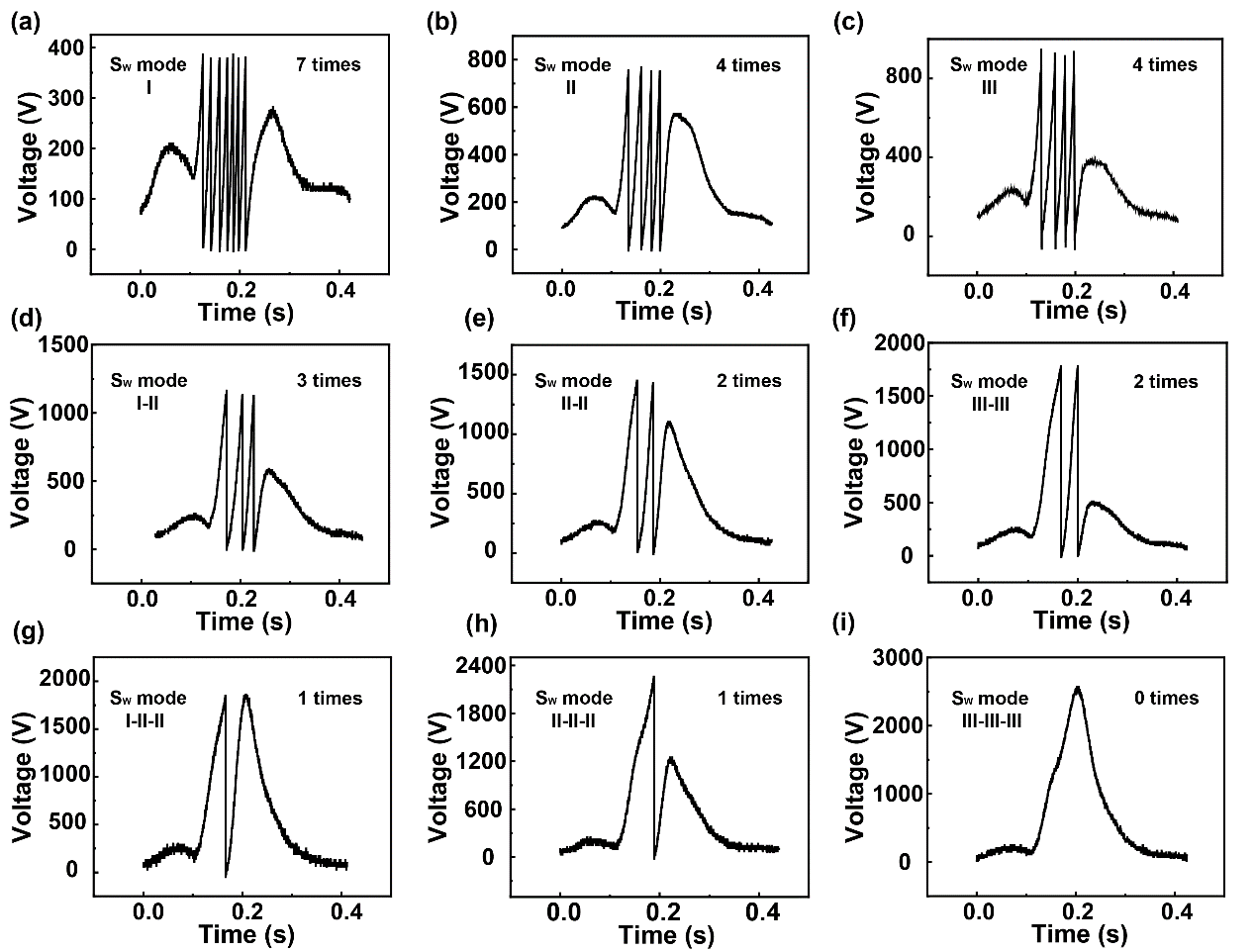


**Fig. S5** The voltage of C_1_ in UV-PMS with different SW modes. Where (a) corresponds to mode I. (b) corresponds to mode II. (c) corresponds to mode III. (d) corresponds to mode I-II. (e) corresponds to mode II-II. (f) corresponds to mode III-III. (g) corresponds to mode I-II-II. (h) corresponds to mode II-II-II. (i) corresponds to mode IIII-III-III.


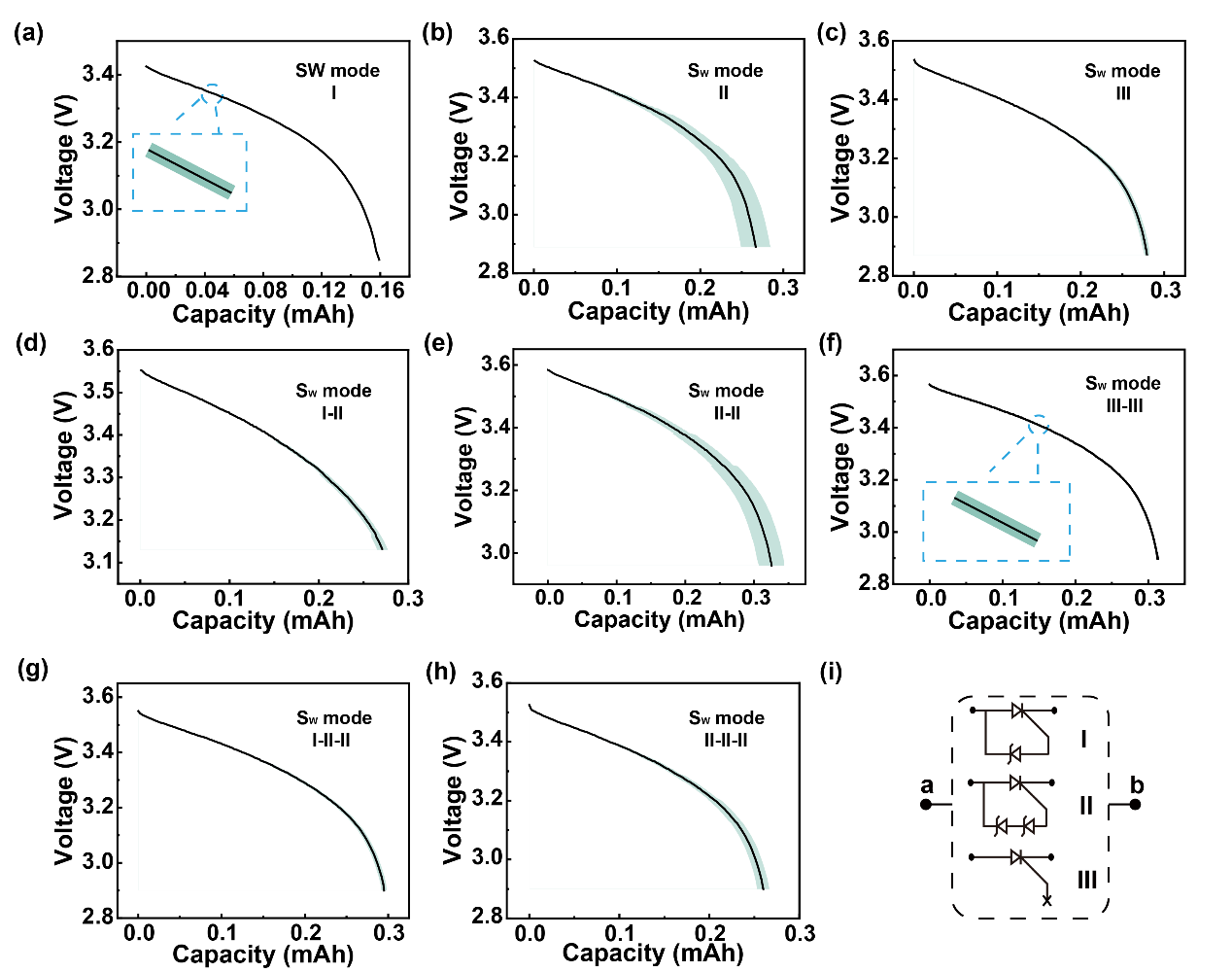


**Fig. S6** The discharge voltage-capacity variation curve and error interval of LIR1220 after charged by TENG through UV-PMS with different SW modes for one hour. Where (a) corresponds to mode I. (b) corresponds to mode II. (c) corresponds to mode III. (d) corresponds to mode I-II. (e) corresponds to mode II-II. (f) corresponds to mode III-III. (g) corresponds to mode I-II-II. (h) corresponds to mode II-II-II. (i) Three basic modes of SW.


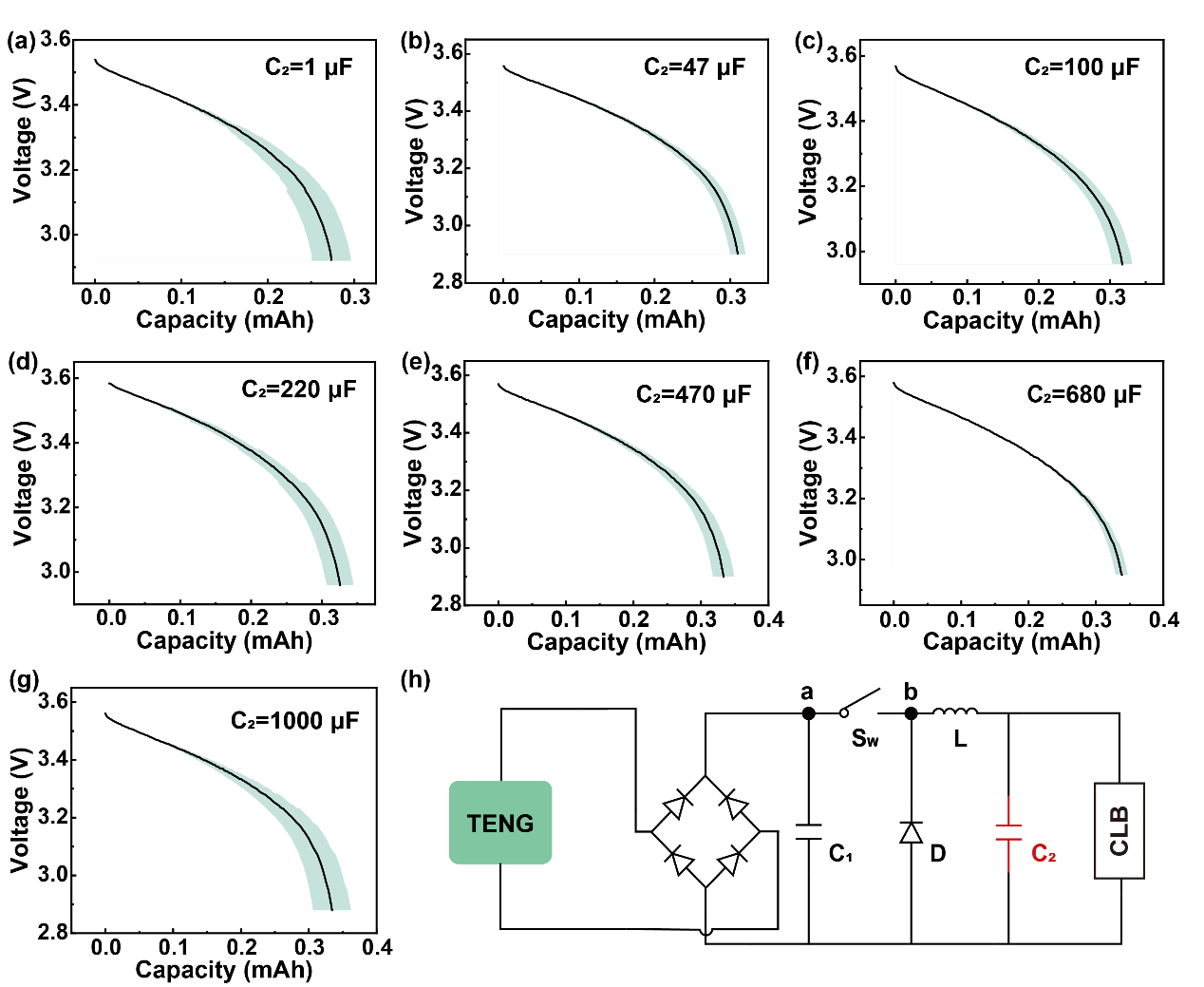


**Fig. S7** The discharge voltage-capacity variation curve and error interval of LIR1220 after charged by TENG through UV-PMS with different capacitors C_2_ for one hour. Where (a) corresponds to C_2_=1 μF. (b) corresponds to C_2_=47 μF. (c) corresponds to C_2_=100 μF. (d) corresponds to C_2_=220 μF. (e) corresponds to C_2_=470 μF. (f) corresponds to C_2_=680 μF. (g) corresponds to C_2_=1000 μF. (h) The circuit of UV-PMS.


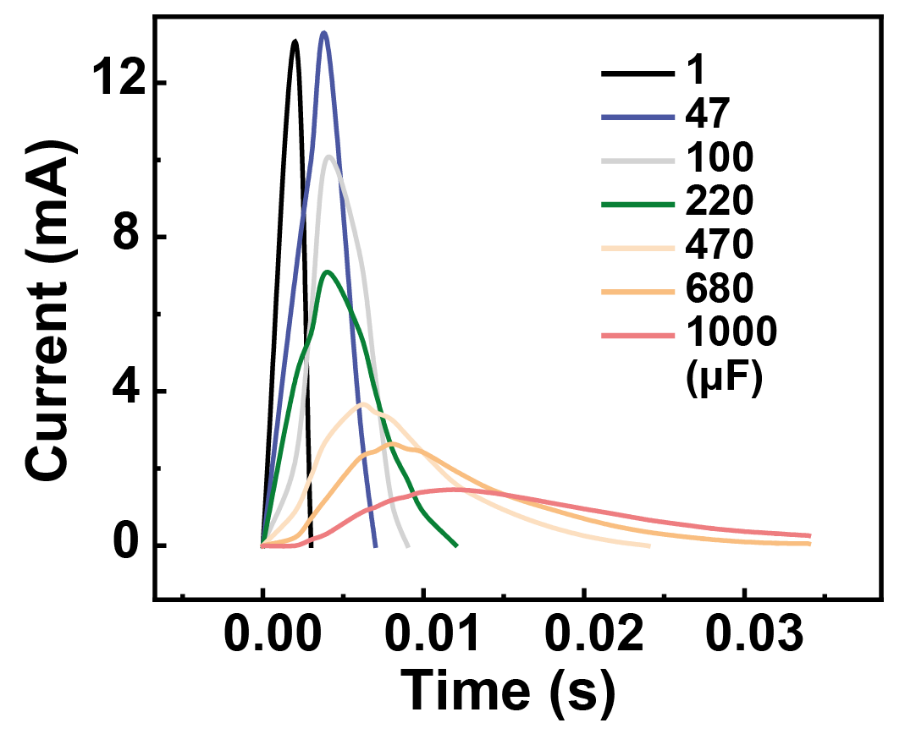


**Fig. S8** Details of peak current width variation for states with different values of C2.


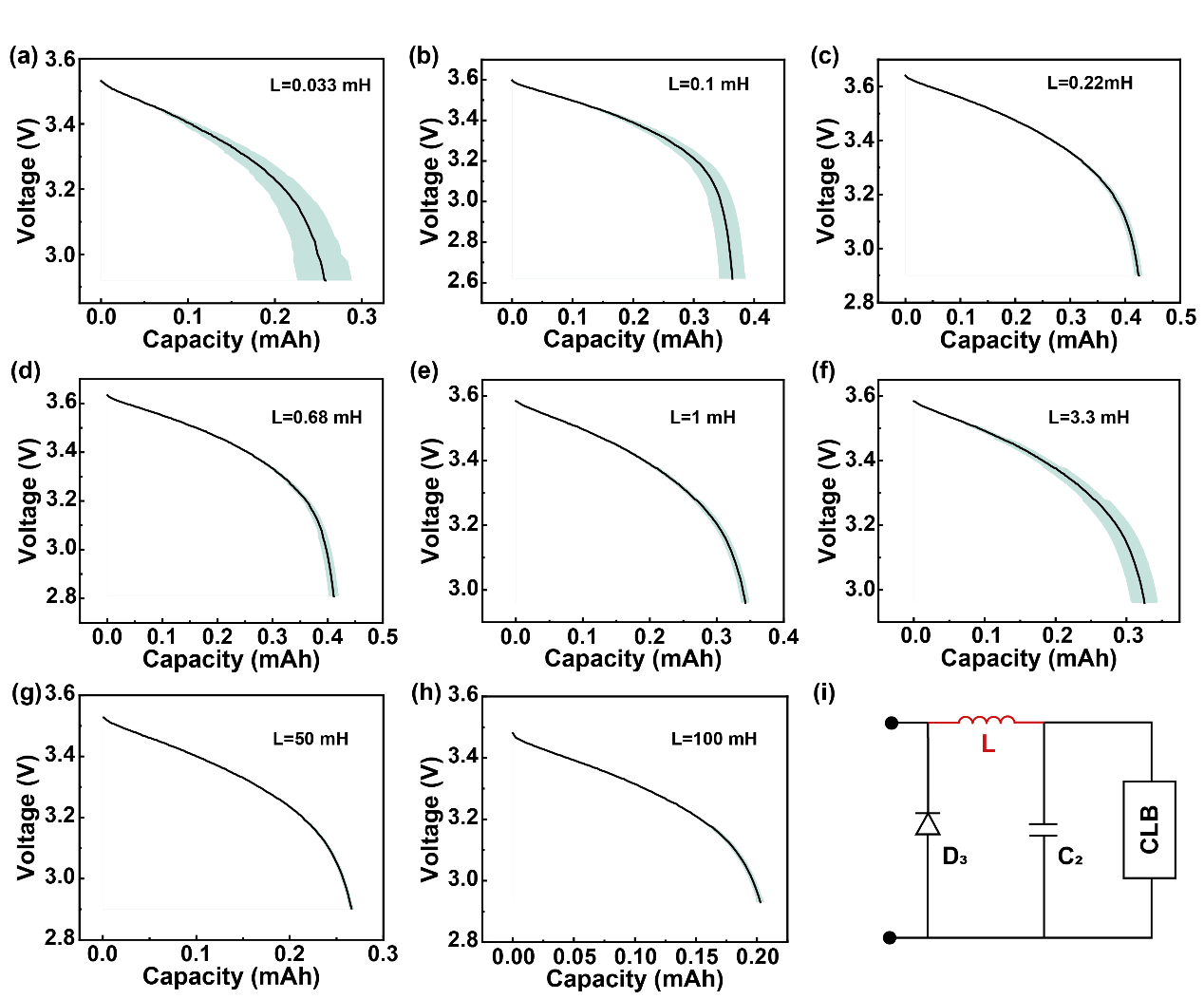


**Fig. S9** The discharge voltage-capacity variation curve and error interval of LIR1220 after charged by TENG through UV-PMS with different inductances L for one hour. Where (a) corresponds to L=0.033 mH. (b) corresponds to L=0.1 mH. (c) corresponds to L=0.22 mH. (d) corresponds to L=0.68 mH. (e) corresponds to L=1 mH. (f) corresponds to L=3.3 mH. (g) corresponds to L=50 mH. (h) corresponds to L=100 mH. (i) Part of the circuit of UV-PMS.


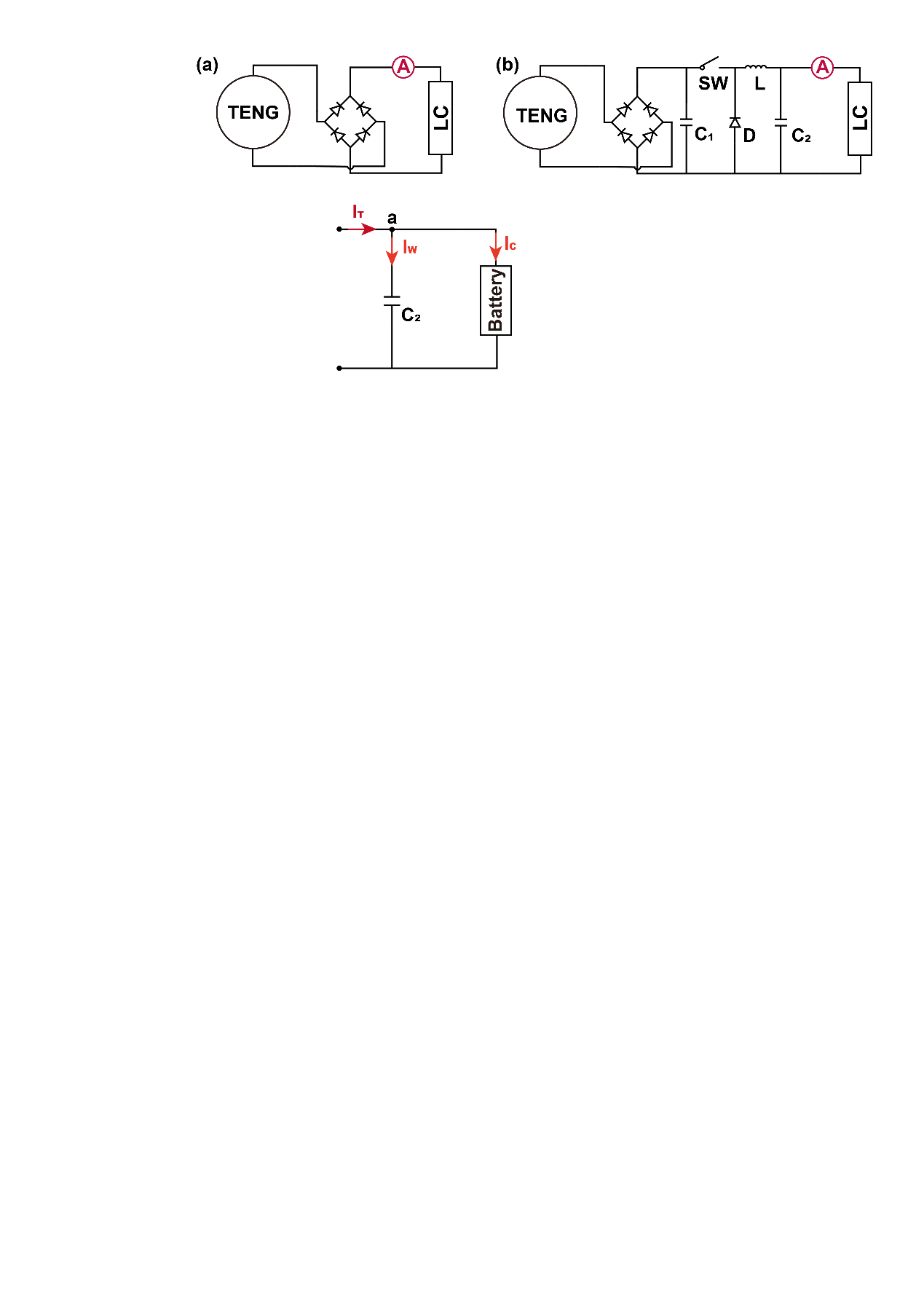


**Fig. S10** The charging current measurement circuit. (a) TENG charge LC directly. (b) TENG charge LC with UV-PMS.


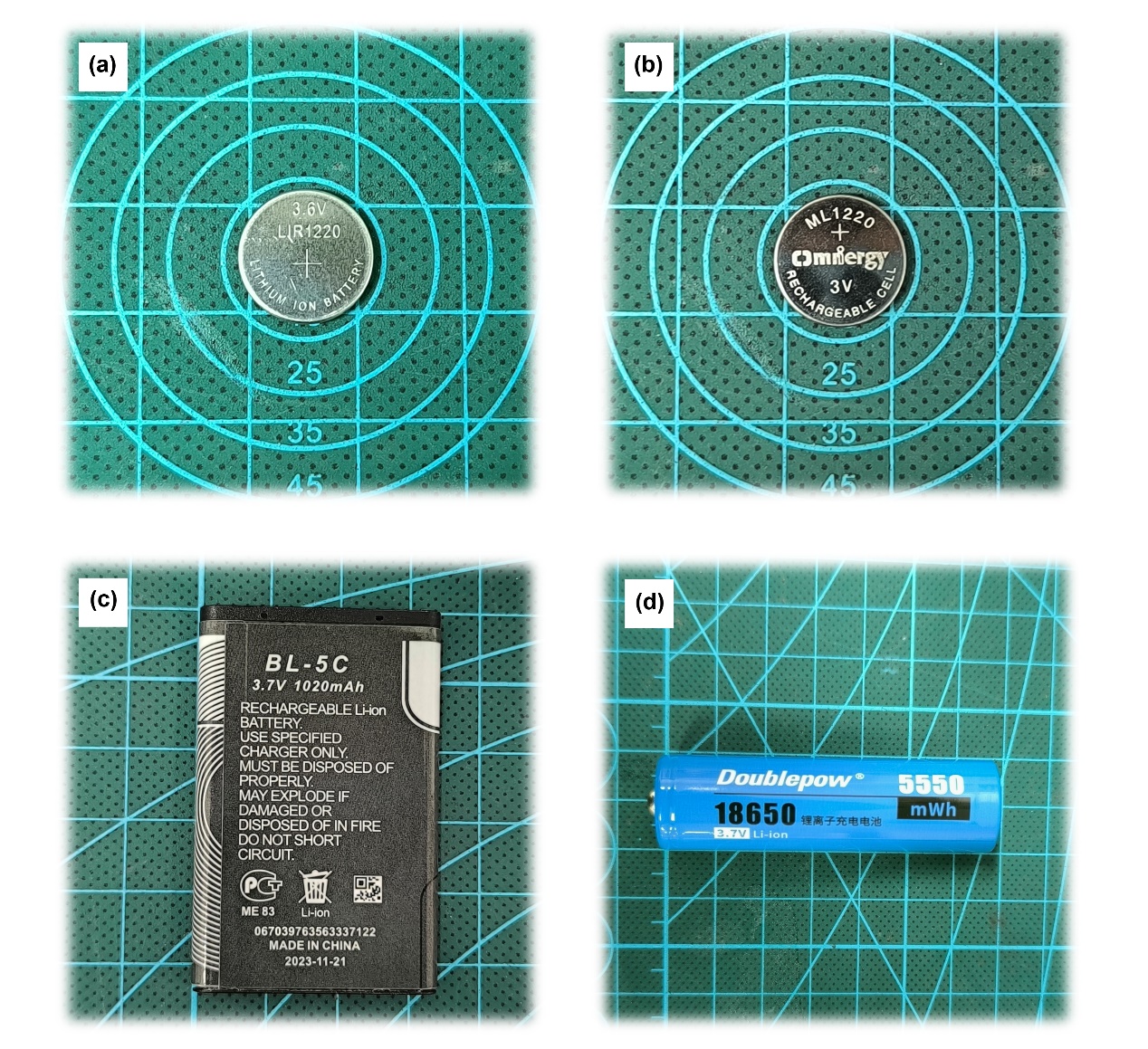


**Fig. S11** Photos of batterie (a) LIR1220, (b) ML1220, (c) BL-5C and (d) 18650.


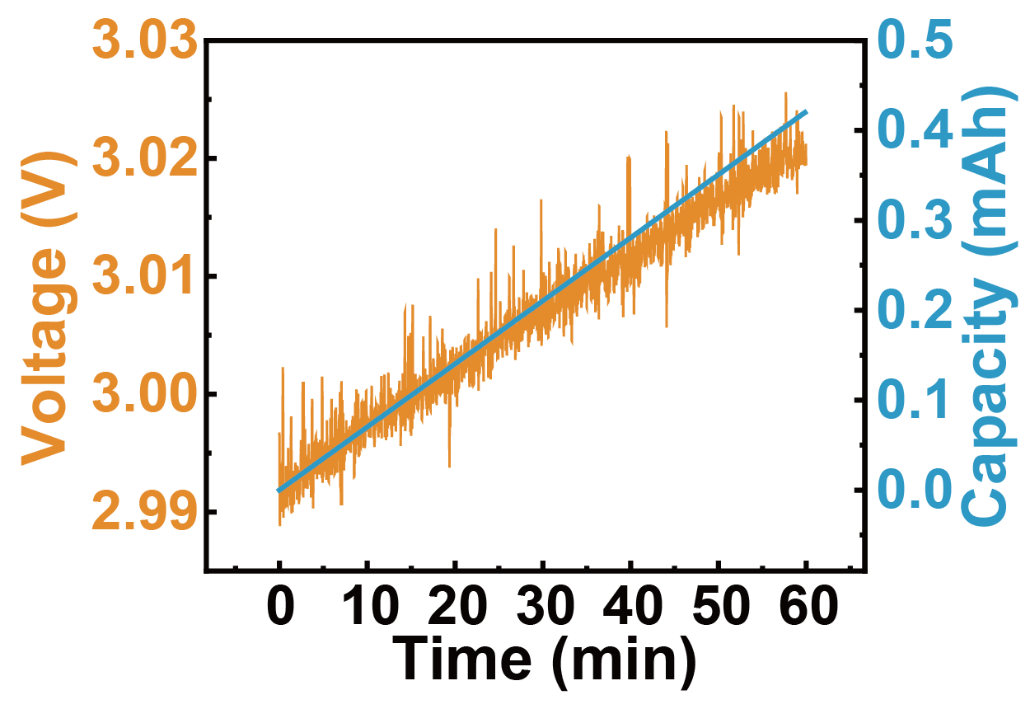


**Fig. S12** The voltage-time variation during charging process and the discharging capacity-time variation curve after charged by TENG of BL-5C LC.


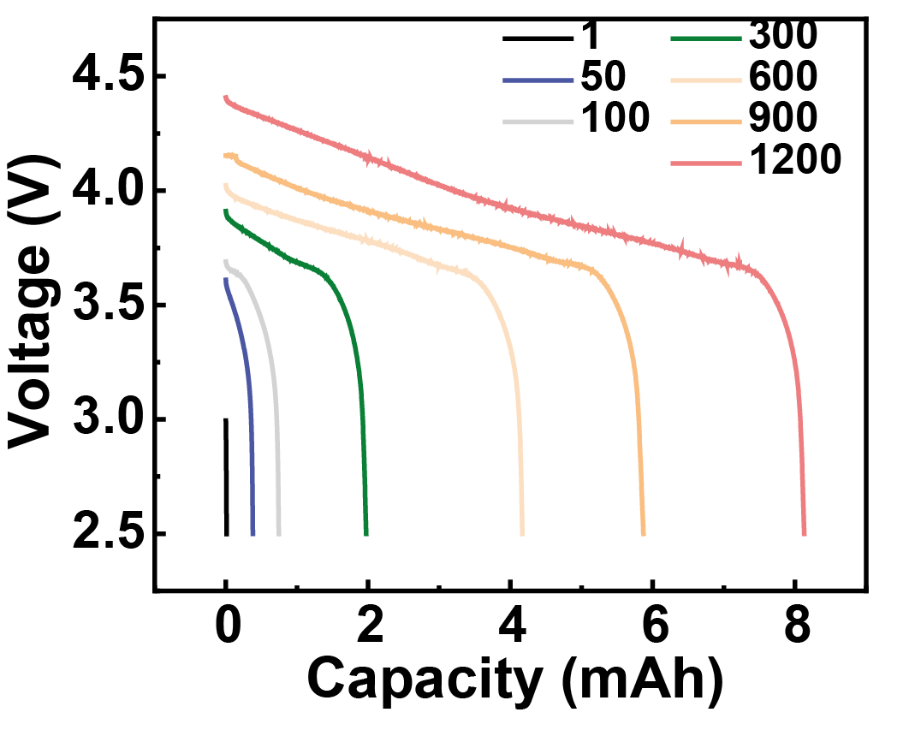


**Fig. S13** The voltage-capacity variation curves of LIR1220 LC during discharging process after different charging durations.


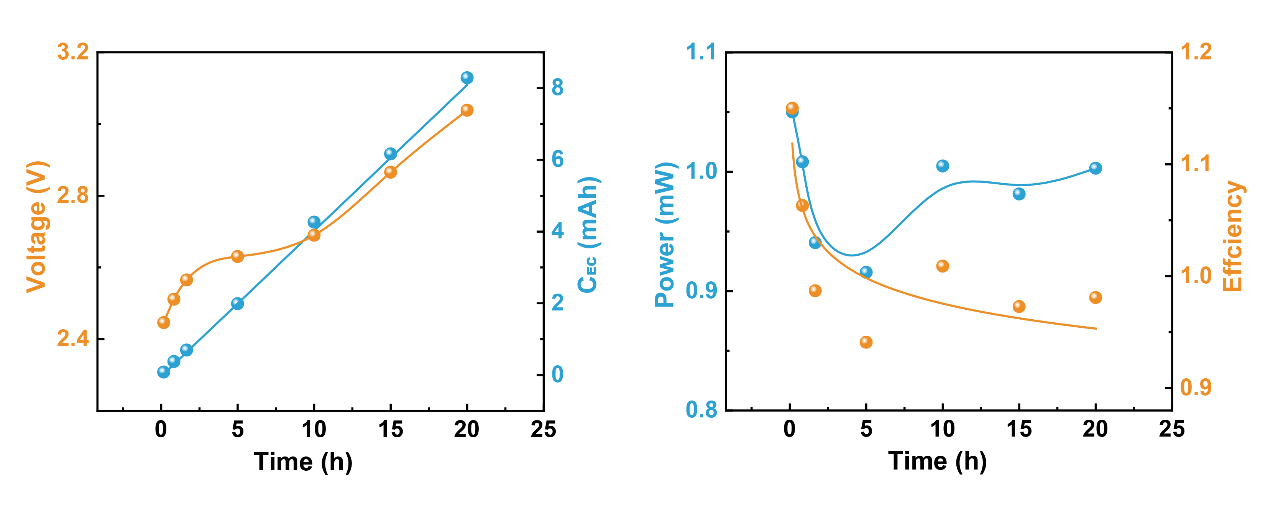


**Fig. S14** Charging characteristics of ML1220 LC. (a) Characteristics of the voltage variation of ML1220 LC charging by this charging solution as well as the variation of the ECC. (b) Charging power variation characteristics of this charging solution for ML1220 LC charging as well as the variation characteristics of charging efficiency.


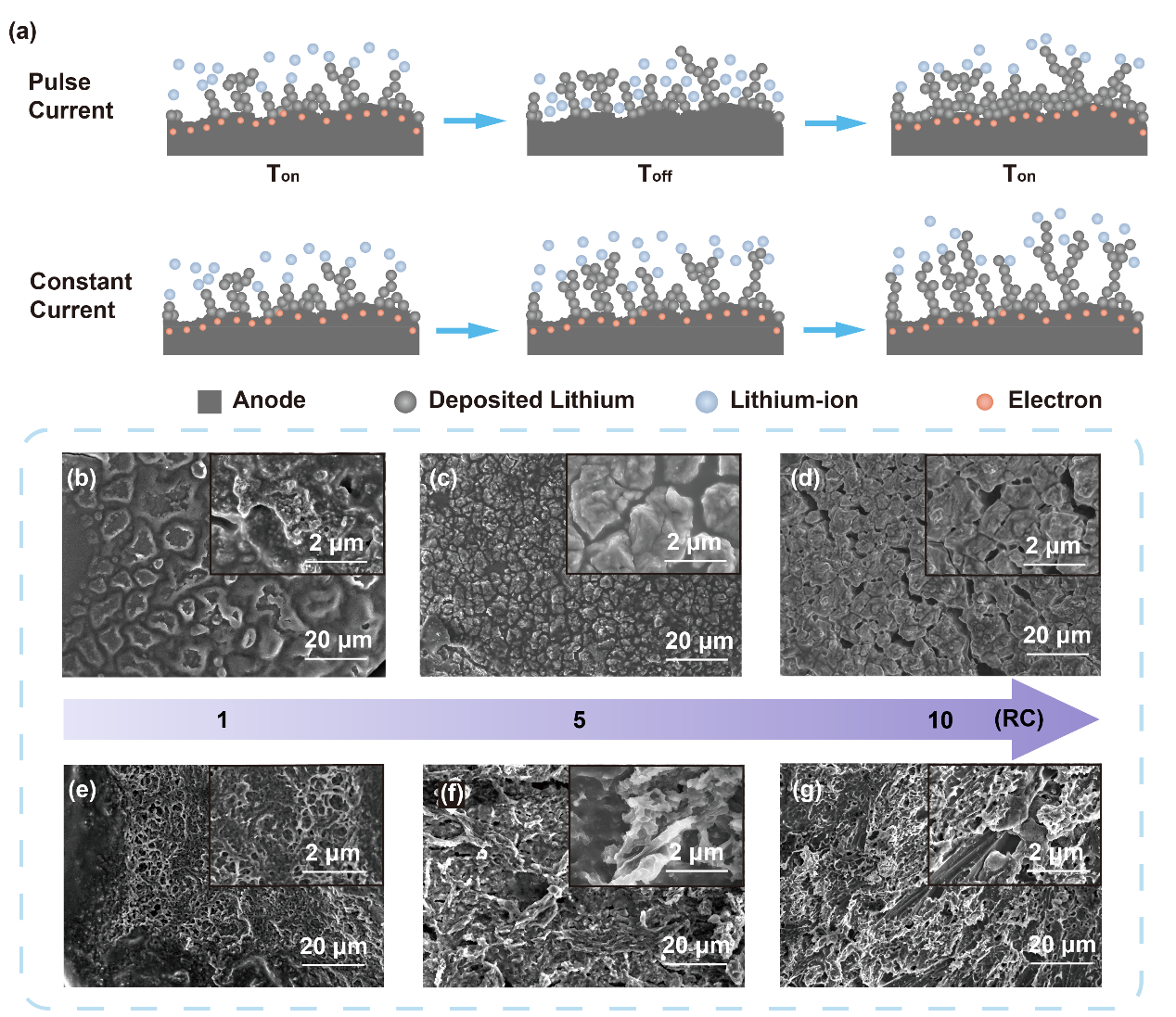


**Fig. S15** Scanning electron microscope (SEM) images of ML1220 LC anode. (a) Schematic diagram of the effect of constant charging current versus pulsed form charging current generated by TENG on the surface morphology of the anode of a lithium metal LC. (b-d) Anode surface morphology of ML1220 LCs after 1, 5, and 10 recharge cycles (RCs) using TENG, respectively. (e-g) Anode surface morphology of ML1220 LCs after 1, 5, and 10 RCs using the integral average current of the TENG charging current (417 μA), respectively.
